# Cognitive Function in Patients Undergoing Arthroplasty: The Implications for Informed Consent

**DOI:** 10.4061/2011/346161

**Published:** 2011-06-20

**Authors:** N. Demosthenous, J. P. St Mart, P. Jenkins, A. Chappel, Kenneth Cheng

**Affiliations:** ^1^Glasgow Royal Infirmary, 84-106 Castle Street, Glasgow G4 0SF, UK; ^2^Inverclyde Royal Hospital, Larkfield Road, Greenock PA16 0XN, UK; ^3^Royal Infirmary of Edinburgh, 51 Little France Crescent, Edinburgh EH16 4SA, UK

## Abstract

Obtaining informed consent for an operation is a fundamental daily interaction between orthopaedic surgeon and patient. It is based on a patient's capacity to understand and retain information about the proposed procedure, the potential consequences of having it, and the alternative options available. We used validated tests of memory on 59 patients undergoing lower limb arthroplasty to assess how well they learned and recalled information about their planned procedure. All patients showed an ability to learn new material; however, younger age and higher educational achievement correlated with better performance. These results have serious implications for orthopaedic surgeons discussing planned procedures. They identify groups of patients who may require enhanced methods of communicating the objectives, risks, and alternatives to surgery. Further research is necessary to assess interventions to improve communication prior to surgery.

## 1. Introduction

Good medical practice is a partnership between clinician and patient. It is based on sharing of information, presenting and discussing treatment options, and arriving at an informed decision. Informed consent is obtained only when a patient has received, processed, and retained this new technical information and arrives at a voluntary decision. The UK General Medical Council outlines the fundamentals of consent as listening to and respecting patients' views, discussing and sharing relevant information required to make a decision with patients, and respecting their decision [1].

There are both ethical and legal implications to obtaining consent. Clinicians strive to achieve the best outcomes for their patients. The consent process explains the proposed procedure in simple to understand language, its purpose, and why it is being proposed. Verbal discussion is occasionally reinforced with written information. The aim is to ensure patients understand the benefits and potential risks, whilst considering the possible alternative treatment strategies. This forms a discussion where patients ask questions and seek clarification. For a patient to have capacity to consent they must be able to receive, retain, understand, and process this information. In addition to the legal requirements, the consent process is fundamental to meeting patient's ideas, concerns and expectations of the procedure, and managing outcomes accordingly.

Several studies have shown that retention of medical information by patients is incomplete and decreases with time, both before and after surgery [2–5]. This, therefore, questions the validity of the consent procedure. 

The primary aim of this study was to assess how well patients about to undergo arthroplasty remember a specific list of complications pertaining to their planned procedure. The secondary aim was to use validated neuropsychological tests to describe their ability to learn and retain information. The impact of age, gender, and education on these outcomes was explored to determine if there are particular patient groups that may benefit from measures to enhance learning and recall.

## 2. Patients and Methods

The study was carried out at the Inverclyde Royal Hospital, Greenock, Scotland, which is a district general hospital that provides a range of elective and acute trauma orthopaedic services. The inclusion criteria were patients over eighteen years of age, undergoing primary total hip replacement, total knee replacement, unicompartmental knee replacement, hip resurfacing, hip revision arthroplasty, or knee revision arthroplasty. Patients were excluded if they had any condition impairing memory or communication (dementia, cerebrovascular disease, epilepsy, head injury, dysphasia, or aphasia). Sixty-five patients were considered for inclusion over a six-month time period, and six were excluded for one of the exclusion criteria above. 

The study population, therefore, comprised 59 patients with a mean age of 68.6 years (SD 10.3, range 41 to 87). There were 20 (33.9%) males and 39 females (76.1%). Eleven (19%) patients left full-time education before 14 years of age, 42 (71%) left education between 15 and 18 years of age, and 6 (10%) completed education after 19 years of age. 

Thirty patients underwent total knee replacement, 17 underwent total hip replacement, 7 underwent hip resurfacing, 4 underwent revision total hip replacement, and 1 underwent revision total knee replacement.

The initial interview was approximately two hours long. Neuropsychological tests were administered to measure the patient's ability to receive, store, and recall information delivered verbally. Wechsler's Logical Memory Test [6] (WLMT) measures immediate and delayed recall. A short story was read to the patient who was then asked to recount it immediately, and again after a rest of 30 min. A point was awarded for each specific point the patient recalled. The number of points recalled at each interval constituted the patients score for immediate and then for delayed recall. 

The Rey Auditory Verbal Learning Test [7] (AVLT) was used to measure verbal learning and memory by reading out fifteen words (at a rate of one word per second). The patient was then asked to recall all fifteen words (one point per word, any order is acceptable). This was repeated five times using the same list to assess learning capacity through repetition. Delayed recall was scored after a rest period of 30 minutes when patients were asked to recall the same list of words.

The National Adult Reading Test [8] (NART) was used to assess cognitive function independent of age and cognitive decline, by asking the patient to read fifty phonetically irregular words. For example: “ache, debt, psalm, and aisle.” It estimates premorbid mental ability in adults because vocabulary correlates best with overall ability level and is relatively unaffected by most nonaphasic brain disorders. One point was awarded for each correctly pronounced word.

After the neuropsychological tests, the planned procedure was explained and a list of ten associated risks (Table 1) was discussed. Patients were encouraged to ask questions about the operation, complications, postoperative rehabilitation, and alternative treatment options. At the end of the interview the risks were reiterated and patients asked to remember them, as they would be asked to recall them on admission for surgery (mean time between assessment and surgery = 20 days, range 14 to 28).

The patients were asked to recall the list of complications when they attended hospital for their procedure. One point per complication remembered. This constituted the end of the study for the patient. Patients were then formally consented, that is, the procedure was explained to them again. They were encouraged to ask questions and express any concerns. They were then given reading material to take home that explained the operation, outlined the risks, and discussed postoperative rehabilitation. 

Ethical approval was obtained from the regional research ethics committee (REC).

### 2.1. Statistics

All statistical calculations were performed using Statistical Package for Social Sciences (SPSS), version 17 (SPSS Inc., Chicago, Illinois). The data was tested for normality with the Shapiro-Wilk test. The NART and complication recall were not normally distributed. Means and standard deviations are reported for parametrically distributed data, and medians with interquartile ranges are reported for nonparametric data. Independent *t*-tests were performed to compare means between independent groups. The Mann-Whitney *U* test was used to compare continuous nonparametric data. Where data were repeated in the same patient, such as immediate and delayed recall, a paired *t*-test was used. A repeated measures analysis of variance (ANOVA) was undertaken to analyse the results from the RAVLT test to compare learning of 5 repetitions, and the delayed recall thereafter. Intergroup comparisons were made between groups based on education level, and age was controlled for as a covariate. Bonferroni corrections were applied to posthoc tests. A level of significance of *P* < .05 was chosen to reject null hypotheses.

## 3. Results

**Figure 1:**
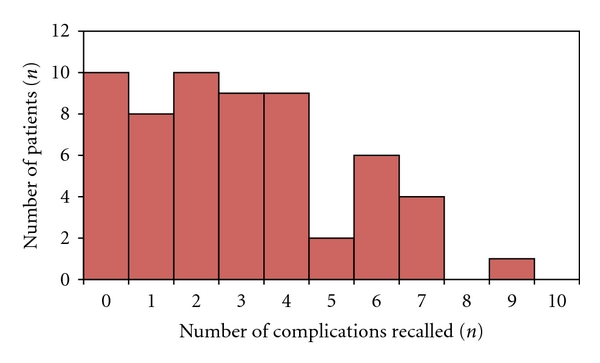
Histogram of number of complications recalled at time of admission for procedure.

Patients recalled a median three complications (IQR 1 to 4) when they attended for their procedure out of 10 that had been outlined at the prior assessment (Table 2) (Figure 1). There was no difference in complication recall between genders (MWU; *P* = .374). There was a negative correlation between complication recall and age (Spearman rho = −0.345, *P* = .007; Table 3). Patients who left full time education after nineteen years of age had a higher level of complication recall (median 3, IQR 1 to 4) compared with those with the least education (leaving full time education before age 14 (median 2, IQR 2 to 2; MWU; *P* = .02). 

**Figure 2:**
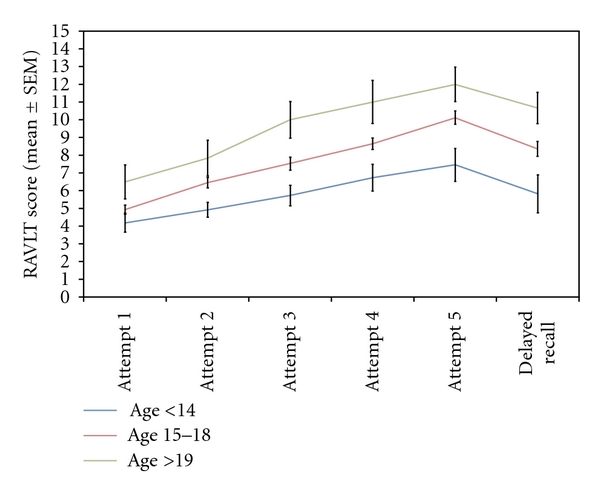
Rey audio-verbal learning test scores during 5 repetitions, and then after a delay, grouped by age left full-time education.

Both the immediate and delayed recall components of the WLMT correlated negatively with age (Table 3). Delayed recall was less than immediate recall (mean difference = 1.6, 95% CI 0.5 to 1.8, *P* = .001). The RAVLT demonstrated increasing scores from attempt 1 to attempt 5 through a decline to delayed recall, corrected for age and education level (ANOVA *P* = .011; Figure 2). Between subject tests demonstrated significant influence of age (*P* = .004) and education (*P* = .039) on RAVLT. There was no interaction of age (*P* = .318) or education (*P* = .229) with RAVLT over time. The median NART score was 31 (IQR 20 to 40). There was no significant difference between gender and any neuropsychological score. 

All scores correlated positively with the number of complications the patients recalled at admission from the assessment (Table 3).

## 4. Discussion

This study provides important quantitative data about the cognitive abilities of a group of patients undergoing arthroplasty. This is particular important as the transmission, receipt, and storage of information about the procedure is vital to both the legal consent process, and in the patient's satisfaction with the outcome of the procedure by managing expectations. This study confirms that the ability to remember complications is poorer as age increases. Patients only recall 3 out of 10 major possible complications, even when specifically told they should remember them. It also shows that neuropsychological tests of memory correlate with the ability to recall this complication list. All patients showed the ability to learn; however, the younger cohort out performed their peers in the AVLT test. This suggests that older patients may need further repetition of the information or different techniques for consolidating information. 

There was a correlation between the number of complications recalled and patients' education level, with those leaving school over the age of 19 years, performing better. This can either be interpreted as a more intelligent subpopulation performing better, or what is more likely, better access to education, that is, the availability of further education, resulting in improved recall of complications. Higher education teaches students how to process information, understand it and recall this at a later date. We believe it is this framework for understanding that has led to better complication recall in our set of patients who left school over the age of 19. With an increasing number of the population pursuing higher education, this factor may be less important in the generation of patients undergoing arthroplasty to come. 

This paper is unique in applying neuropsychological tests to a group of orthopaedic patients undergoing arthroplasty. These tests are validated and objective, and have been in widespread use over many years. They provide a baseline for future research that may assess the effect of interventions to improve the giving and remembering of information. The testing situation was designed to mirror a clinical situation where information is mainly given verbally through a one-to-one discussion between clinician and patient. 

The weaknesses of this study include the fact that the scenario of neuropsychological cognitive testing does not fully match a typical clinic. In a clinic, pressure of time or commitments, combined with a noisy environment may reduce the ability of the clinician to communicate information and check that it has been properly retained and understood. It also cannot assess the instinctive changes in communication style that a clinician may make when faced with a patient they perceive as having difficulties understanding proceedings. Patients may also undertake their own research using the internet or discussing the procedure with their general practitioner. The combination of different tests may lead to fatigue towards the end of testing and poorer accuracy of the tests performed last. 

Our results are in accordance with Hutson and Blaha study [3] 20 years ago where patient recall of preoperative instruction for informed consent for an operation was poor. It is clear that newer patient education techniques are needed to prepare patients for their operation. Information should be given to patients verbally, but also supplemented with written information that they can read and refer to later. The information should be given at several different points, such as initial clinic appointment, preadmission, prior to surgery, and postoperatively [2]. Lee et al. [10] compared the effectiveness of media-based patient education about anaesthetic by showing patients a video preoperatively about what to expect perioperatively. They found that patients in the video group were more likely to answer all knowledge questions correctly compared with patients with no intervention. Further more, the level of knowledge about pain management was higher in the video group compared with patients with no intervention. Although they did not look at specific demographics such as age and level of education, this may prove an effective tool in educating arthroplasty patients about their operation. 

Clinicians should be aware of negative effects of increasing age or younger age at leaving full-time education, and allow extra measures such as increased repetition, the provision of easy to understand written material, and the checking of recall prior to surgery and immediately after. Clinicians should also be aware of particular groups where memory is impaired through disease (cerebrovascular, dementia, etc.) and the ability to consent to the procedure should be fully explored. 

Future research should be performed to investigate how verbal and written information may be improved to improve the consent process, as well as newer methods such as online tutorials, videos, and perhaps discussion forums for patients. This should be through prospective randomised trials.

## 5. Conclusion

The doctor-patient dynamic is based on honesty and openness. Patients require capacity to consent for a procedure. Capacity relies on a patient's ability to process and retain vital information regarding a procedure. We have shown that this retention of information is, at least in part, dependent on both patient age and education levels. This is increasingly important in orthopaedic patients undergoing arthroplasty because of an aging population, and therefore questions their ability to provide consent. Consent may still be possible if interventions are used to aid the transmission and recall of the benefits and risks of surgery.

## Figures and Tables

**Table 1:** Risks associated with knee or hip arthroplasty [9].

Risks
Blood clot in legs (deep venous thrombosis)
Blood clot in lungs (pulmonary embolism)
Bleeding (either small or large amount requiring blood
transfusion)
Pain (short term or chronic)
Implant wear or loosening
Altered leg length
Joint dislocation
Infection of new joint or surgical site
Nerve damage
Altered wound healing (keloid scar)

**Table 2:** Results of Weshchler Logical Memory Test (WLMT), Rey Audio-Verbal Learning Test (RAVLT), National Adult Reading Test (NART), and recall of complication list at time of admission.

		Test score
WLMT (mean, SD)	Immediate	12.2 (4.3)
	Delayed (30 mins)	11.0 (4.8)

RAVLT (mean, SD)	Attempt 1	4.9 (1.8)
	Attempt 2	6.3 (2.0)
	Attempt 3	7.4 (2.5)
	Attempt 4	8.5 (2.5)
	Attempt 5	9.8 (2.8)
	Delayed (30 mins)	8.1 (3.1)

NART (median, IQR)		31 (20 to 40)

Complication recall (median, IQR)		3 (1 to 4)

**Table 3:** Correlation of cognitive testing with age and complication recall.

		Correlation coefficient age (Spearman *ρ*, *P* value)	
		Age	Complications

WLMT	Immediate	−0.38, *P* = .003	0.34, *P* = .009
	Delayed	−0.42, *P* = .001	0.39, *P* = .003

RAVLT	Delayed	−0.45, *P* < .001	0.38, *P* = .003

NART		−0.14, *P* = .296	0.40, *P* = .002

Complications		−0.345, *P* = .007	N/A

## References

[1] http://www.gmc-uk.org.

[2] Lavelle-Jones C, Byrne DJ, Rice P, Cuschieri A (1993). Factors affecting quality of informed consent. *British Medical Journal*.

[3] Hutson MM, Blaha JD (1991). Patients’ recall of preoperative instruction for informed consent for an operation. *Journal of Bone and Joint Surgery A*.

[4] Askew G, Pearson KW, Cryer D (1990). Informed consent: can we educate patients?. *Journal of the Royal College of Surgeons of Edinburgh*.

[5] Herz DA, Looman JE, Lewis SK (1992). Informed consent: is it a myth?. *Neurosurgery*.

[6] Wechsler D (1997). *Weschsler Adult Intelligence Scale-III*.

[7] Lezak MD, Howieson DB, Loring DW, Hannay HJ, Fischer JS (1995). *Neuropsychological Assessment*.

[8] Nelson HE (1982). *The National Adult Reading Test (NART): Test Manual*.

[9] http://www.orthoconsent.com/body2.asp?BodyPartID=4.

[10] Lee A, Chui PT, Gin T (2003). Educating patients about anesthesia: a systematic review of randomized controlled trials of media-based interventions. *Anesthesia and Analgesia*.

